# Gender Inequalities of Health and Quality of Life in Informal Caregivers in Spain: Protocol for the Longitudinal and Multicenter CUIDAR-SE Study

**DOI:** 10.2196/58440

**Published:** 2024-07-02

**Authors:** María del Mar Garcia-Calvente, Diana Juanita Mora, María Del Río-Lozano

**Affiliations:** 1 Escuela Andaluza de Salud Pública Granada Spain; 2 Instituto de Investigación Biosanitaria de Granada Granada Spain; 3 École des Hautes Études en Santé Publique Rennes France

**Keywords:** caregivers, gender equity, sex differences, health status, quality of life, longitudinal studies, multicenter studies

## Abstract

**Background:**

The aging population and increased disability prevalence in Spain have heightened the demand for long-term care. Informal caregiving, primarily performed by women, plays a crucial role in this scenario. This protocol outlines the CUIDAR-SE study, focusing on the gender-specific impact of informal caregiving on health and quality of life among caregivers in Andalusia and the Basque Country from 2013 to 2024.

**Objective:**

This study aims to analyze the gender differences in health and quality of life indicators of informal caregivers residing in 2 Spanish autonomous communities (Granada, Andalusia, and Gipuzkoa; Basque Country) and their evolution over time, in relation to the characteristics of caregivers, the caregiving situation, and support received.

**Methods:**

The CUIDAR-SE study uses a longitudinal, multicenter design across 3 phases, tracking health and quality of life indicators among informal caregivers. Using a questionnaire adapted to the Spanish context that uses validated scales and multilevel analysis, the research captures changes in caregivers’ experiences amid societal crises, notably the 2008 economic crisis and the COVID-19 pandemic. A multistage randomized cluster sampling technique is used to minimize study design effects.

**Results:**

Funding for the CUIDAR-SE study was in 3 phases starting in January 2013, 2017, and 2021, spanning a 10-year period. Data collection commenced in 2013 and continued annually, except for 2016 and 2020 due to financial and pandemic-related challenges. As of March 2024, a total of 1294 participants have been enrolled, with data collection ongoing for 2023. Initial data analysis focused on gender disparities in caregiver health, quality of life, burden, perceived needs, and received support, with results from phase I published. Currently, analysis is ongoing for phases II and III, as well as longitudinal analysis across all phases.

**Conclusions:**

This protocol aims to provide comprehensive insights into caregiving dynamics and caregivers’ experiences over time, as well as understand the role of caregiving on gender inequality in health, considering regional variations. Despite limitations in participant recruitment, focusing on registered caregivers, the study offers a detailed exploration of the health impacts of caregiving in Spain. The incorporation of a gender perspective and the examination of diverse contextual factors enrich the study’s depth, contributing significantly to the discourse on caregiving health complexities in Spain.

**International Registered Report Identifier (IRRID):**

DERR1-10.2196/58440

## Introduction

### Caregiving in the International and Spanish Context

In recent decades, Spain has witnessed significant demographic changes, marked by an aging population and a notable prevalence of disability among older adults [1]. With the highest percentage of older adults in the Organisation for Economic Co-operation and Development (OECD), at 5% older than 80 years, Spain’s aging population is expected to double by 2050, leading to a 6-fold increase in public spending on long-term care (LTC) [2]. In addition, Spain is home to 4,380,000 people with disabilities as of 2020 where over 2 million receive personal or supervised care [3].

In the context of this study, informal care is defined as care activities carried out by people in the dependent person’s immediate environment (relatives, friends, or neighbors), in a nonprofessional manner and who do not receive remuneration for the work they do (although they may sometimes benefit from financial benefits). Informal care is a pillar of LTC systems among OECD countries [4]. It is estimated that across 23 OECD countries, around 14% of people over 50 provide informal care on a daily or weekly basis [4]. In the European Union, it is estimated that between 12% and 18% of the adult population aged 18-75 years provide informal care on a daily or weekly basis [4]. Informal caregivers in Spain formed 12.4% of working people aged 18-64 years [5]. Spain’s LTC landscape encompasses family-centric caregiving, often supplemented by domestic service employment, particularly among immigrant women [6]. Regional variations in this trend stem from differing government priorities, provision levels, labor market dynamics, and caregiving cultures.

### Gender Differences in Informal Caregiving and Its Impact on Health and Quality of Life

The nature of informal caregiving includes its lack of visibility and social recognition. This informal caregiving is tied to personal relationships, domestic in nature, and predominantly carried out by women, which leads to being undervalued socially and economically [7]. Most of the informal care across 25 OECD countries is performed by women, where women represent 60% of daily caregivers [4]. The share of women caregivers is higher in South European OECD countries, with women being 76% of daily informal caregivers in Spain [4]. Gender roles are key, with women in Spain mainly taking on caregiving responsibilities, in addition to handling domestic tasks and sharing close kinship ties with those they care for [8-10].

Gender disparities extend to the type and amount of care provided. Women caregivers are more likely to provide personal care, companionship, and monitoring, often in intensive ways, while juggling family and work obligations, in comparison to men [11]. Despite its societal importance, caregiving can negatively impact caregivers’ physical and mental health due to chronic stress from the physical and emotional burden [11-14]. Factors such as the cared-for person’s disability type and behavioral issues, especially dementia, alongside the care type and intensity, contribute to caregiver stress and health issues [11-19]. These effects on health can manifest as poor physical health, mental health challenges, unhealthy habits, and even premature death [11-19]. Variables like gender, age, socioeconomic status, and social support play a role in moderating these effects [11]. However, population-based studies show that almost one-third of caregivers do not experience negative impacts, particularly in the early caregiving stages [20].

Caregiving also affects other quality of life dimensions. It often reduces employment opportunities, leads to fewer work hours, and increases temporary work, affecting women more than men, and potentially increasing poverty risk [9,21]. Survey data in Spain highlight a higher prevalence of health, professional, economic, and personal problems due to the higher burden of caring [22]. The unequal burden on women’s daily lives leads to higher stress, overload, and risk factors for their health [23-26]. Various studies confirm this pattern, indicating more psychiatric morbidity, depression, and poorer health perception among women caregivers [22-26]. However, while existing research underscores these trends, there is a need for further exploration through quantitative and qualitative methods [27].

### Need for Follow-Up and Longitudinal Studies

While existing evidence highlights caregiving’s impact on health and quality of life, many studies experience limitations like small sample sizes and cross-sectional designs, often lacking control over confounding factors, such as education or socioeconomic status. To comprehensively address these issues, longitudinal studies are imperative. These studies not only counter cross-sectional biases, such as the “healthy caregiver” bias, but also track caregivers’ health and quality of life changes over the caregiving journey [28]. Additionally, a deeper understanding of varied caregiving experiences and their effects on caregivers’ health is crucial [19]. Longitudinal research on these aspects remains limited in the Spanish context, and the factors influencing the impact of caregiving over time still require in-depth exploration.

### The Importance of Crisis Contextualization

In recent decades, women’s engagement in the workforce and formal care services for dependents have surged, yet the ongoing crisis is bucking this trend by shifting caregiving responsibilities back to households [29]. The reduction in formal care services may intensify informal caregiving, potentially overburdening caregivers. With escalating unemployment, particularly affecting women, the adverse impacts of caregiving on employment and finances will worsen, especially in a landscape of diminished public support services. This scenario is expected to detrimentally affect caregivers’ health and well-being, especially among women [30]. Vulnerable groups like older adults, minors, dependents, and women will bear amplified health-related repercussions during crises [31]. Effective support networks, both formal and informal, can mitigate these effects, highlighting the importance of evaluating how informal networks, mainly family-based, impact women’s health and quality of life as primary caregivers [32]. The study at hand was initiated in 2013, amidst the repercussions felt in the following years after the 2008 economic crisis which was then further compounded by the COVID-19 pandemic [33]. These 2 crises set the contextual background for the study, and therefore it is important to acknowledge these significant events’ relevance in the realm of caregiving, and subsequently, in the study.

### The CUIDAR-SE Study

CUIDAR‐SE is the acronym derived from the terms care and follow‐up (“cuidar” and “seguimiento” in Spanish) and is the abbreviated name of the project titled “Longitudinal study of women and men caregiver’s health and quality of life in Andalusia and the Basque Country.” This study was designed to analyze the effects of caregiving on different aspects of life, including health, in a sample of women and men providing informal care in Spain.

Its longitudinal analysis of quantitative data, with data collected 3 times every 12 months, in each of the 3 phases, allows the study to capture caregivers’ experiences during both crises and their ongoing repercussions.

The CUIDAR-SE study also incorporates a gender approach in all its aspects, meaning not only in analysis with sex-disaggregated data but also in its design. The study was designed to conduct comparisons between women and men. This gender perspective has not been found as a focus in previous studies and therefore illustrates the study’s importance.

Furthermore, this study is multicentric in nature. There are notable differences between the 2 contexts at hand. Andalusia (south of Spain) has been characterized in the last decades by being a “familistic regime with subsidized public support,” with support primarily focused on economic benefits. Whereas in the Basque Country (north of Spain), following an “optional familist” model, other social protection services have been further developed to support care and there is a high participation in the domestic market [6,7]. In 2023, the public dependency system, in both regions, has granted more services to support care than economic benefits [34].

As the economic crisis impacts all regions, Andalusia appears to experience more pronounced effects than the Basque Country, potentially accentuating these disparities further. Consequently, this investigation is apt for addressing these dynamics in both contexts. The multidimensional approach facilitated by a multicenter design enriches the study’s uniqueness and significance.

### Objective

To analyze the gender differences in health and quality of life indicators of informal caregivers residing in 2 Spanish autonomous communities (Granada, Andalusia, and Gipuzkoa; Basque Country) and their evolution over time, in relation to the characteristics of caregivers, the caregiving situation, and the support received.

The CUIDAR-SE study is divided into 3 phases, currently in the CUIDAR-SE III phase. Each phase has its respective specific objectives demonstrated in Textbox 1.

**Specific objectives of the 3 phases of CUIDAR-SE study.**

**Phase I**

- Describe the characteristics of the care situation and the indicators of health and quality of life at different times in the care process.
- Analyze the changes (evolution) produced in the health and quality of life of informal caregivers over time.
- Study the differences in the health and quality of life of women and men informal caregivers and their associated factors in relation to the characteristics of the informal caregivers.
- Compare the health and quality of life of women and men caregivers, as well as its evolution, in 2 different national contexts: Granada and Gipuzkoa.

**Phase II**

- Study the evolution of health-related quality of life and the burden of caregivers, based on the informal and formal support (financial benefits and services) received, analyzing the differences between women and men caregivers.
- Estimate the economic value of informal care time and its evolution during the care process, as well as the differences in this value depending on the sex of the caregivers, the support received, and the characteristics of the care provided (tasks and intensity).
- Compare the 2 provinces studied, the evolution of health-related quality of life and the burden of caregivers based on the formal and informal support received, and the economic value of care.

**Phase III**

- Analyze the evolution of perceived health, morbidity in chronic pathologies, and the mental health of women and men caregivers, based on the characteristics of the care provided and the support they receive.
- Know the differences between women and men informal caregivers in the use of health services for their own health needs, their medication use, and the evolution at different moments of the care process.
- Analyze the evolution of the problems derived from care, in women and men caregivers, related to aspects of health, work and professional, economic, leisure, free time, and family life, depending on the characteristics of the care they provide and the support they receive.
- Study the support needs experienced by women and men caregivers at different times in the care process, depending on the characteristics of the care they provide and the formal and informal support they receive.
- Compare Granada and Gipuzkoa the health indicators studied, use of health services, medication use, and problems and support needs exposed in the previous objectives.

## Methods

### Design

This study is a multicenter (Granada, Andalusia, and Gipuzkoa; Basque Country) longitudinal repeated measures study where the caregiver population is followed up at 3 moments in time known as waves, each 12 months in duration, for each phase (I, II, and III) of the CUIDAR-SE study that initiated in 2013 and is ongoing.

### Participants

Participants are composed of all persons aged 18 years or older living in family dwellings in the provinces of Granada and Gipuzkoa who provide unpaid care for a person in a situation of dependency and are co-inhabiting with the individual or not, among other criteria (Textbox 2).

Agreements with the institutions were made to ensure the availability of data on caregivers. From the registries listed (Textbox 2), participants for each phase of the CUIDAR-SE study have been selected using multistage randomized cluster sampling using municipalities as primary units, census sectors as secondary units, and caregivers as final units. The initial sample size calculations were 1180 people, however, due to resource limitations, the sample size was reduced, remaining an equal sample size in terms of sex and province (Table 1).

For each subsequent phase, participants who expressed their willingness to collaborate in future phases were retained. The ongoing follow-up involves the same initial cohort of participants, with the exception of additional individuals recruited during the inception of CUIDAR-SE II to address attrition observed at the conclusion of CUIDAR-SE I.

**Inclusion and exclusion criteria for participants in the CUIDAR-SE study.**

**Inclusion criteria**

- Registered in the records of caregivers available in the health districts of Granada (Primary Care Health District) or in the Gipuzkoa Provincial Council (Social Services Registry) at the time of the beginning of the phase.

**Exclusion criteria**

- Presents characteristics that prevent answering the questionnaire in its language or a change of address is foreseen that prevents follow-up.

**Table 1:** Sample sizes and sex distribution of the phases of the CUIDAR-SE study (2013-2024).

Phase and waves		Gender		
		Men, n	Women, n	Total, n
**Phase I**				
	2013	265	345	610
	2014	208	259	467
	2015	155	199	354
**Phase II**				
	2017	361	478	839
	2018	259	351	610
	2019	184	254	438
**Phase III**				
	2021	96	165	261
	2022	57	98	155
	2023 ongoing	51	99	150

### Participant Recruitment and Follow-Up

The persons selected are initially contacted by means of a letter by those responsible for the registries, by which they will ask for permission from the persons contacted to participate in the study. Once permission is obtained, a member of the research team contacts the participants to describe the scope and objectives of the study, resolve their doubts, guarantee their participation, and request their consent. At the time of data collection, signed informed consent forms are collected. For those who do not consent, basic information is obtained to characterize their refusal to take part in the study.

Participant follow-up occurs at baseline, at 12 months, and at 24 months, for each phase of the CUIDAR-SE study. No new participants will be included after the start of each wave of the study. Participants may leave the follow-up process due to the following cases: (1) end of the follow-up period, (2) death of the caregiver, (3) definitive cessation of the caregiving situation (death or recovery of the cared-for person or cessation of the caregiver’s role due to other circumstances), (4) relocation of residence outside the provinces of the study, and (5) any other circumstance that makes it impossible to locate the caregiver. The information collected on participants who have left the follow-up will be incorporated into the analysis if they have participated in at least 2 moments of measurement.

### Survey Questionnaire Instrument

The questionnaire developed for the study “Health care in the domestic setting: care and caregivers in Andalusia” by García Calvente, 1999, proved useful to identify the health impact of caregiving tasks and its application in this project is considered very appropriate [10]. This questionnaire was updated and adapted to the objectives of the CUIDAR-SE study, considering, among other things such as context, the perception of the new aids instituted under the development of the Spanish Dependency Law. In addition, the scales EuroQol, the DUKE-UNC of 11 categories, the ZARIT scale of burden, and the GOLDBERG of 12 categories were selected for inclusion in the study questionnaire due to their widespread use in similar research endeavors and validation for use within our study context [35-38].

The finalized questionnaire was piloted during CUIDAR-SE phase I in a sample of 20 participants belonging to both provinces to guarantee its comprehension and adequacy. A summary of the structure of the questionnaire can be found in Table 2. The questionnaire was also later complemented with questions found in the 2008 EDAD; a survey on disability, personal autonomy, and situations of dependency conducted by the National Statistics Institute of Spain [39].

**Table 2:** Structure summary of the finalized questionnaire of the CUIDAR-SE study.

Block	Questionnaire structure	Subsections
I	Household structure and people in care	Section A: Household structure Section B: General characteristics of the care recipient
II	Characteristics and intensity of care	Section C: Type of care (care tasks) Section D: Specific tasks related to the use of health services for the care recipient Section E: Frequency, intensity, and duration of care Section F: Other domestic and care workloads of the caregiver Section G: Burden
III	Support, needs, and demands	Section H: Social support Section I: Informal care support Section J: Formal care support Section K: Needs and demands
IV	General and mental health status	Section L: General state of health Section M: Morbidity, mental health, and medication use Section N: Use of health services Section O: Preventive practices Section P: Healthy habits Section Q: Satisfaction with life
V	Consequences of care on health and quality of life	Section R: Impairment of health Section S: Professional and labor aspects Section T: Economic aspects Section U: Aspects of leisure, free time, and family life Section V: Perception of positive effects of care
VI	Contingent economic evaluation of care	—^a^
VII	Sociodemographic	—

^a^Not available.

### Data Collection

A survey technique is used by conducting a personal interview with the caregiver, using the questionnaire. The questionnaire is applied by means of a personal interview at the caregiver’s home, or, when this is not feasible, in-person at a care center (health or social services center, depending on the province). The interview is conducted by specially trained interviewers from a company specializing in nationwide surveying.

The research team provided training workshops to interviewers at the Andalusian School of Public Health, oriented toward understanding the structure and layout of the questionnaire and to provide context for the questions asked in the questionnaire. There is continual interaction between the research team and the interviewers during fieldwork, establishing a quality control protocol.

### COVID-19 Implications

The collection was not completed in 2020 due to the COVID-19 pandemic, and therefore the first wave of phase CUIDAR-SE III was performed subsequently in 2021, 2022, and is currently ongoing. In-person interviews were replaced with telephone interviews using computer-assisted telephone interviewing in 2021 and onward. Certain variables were eliminated to reduce the length of the telephone questionnaire, and questions regarding COVID-19 were added to explore different dimensions of the COVID-19 pandemic and their impact on self-perceived caregiver health and other aspects of life. This new questionnaire was piloted among 60 members of the current cohort to ensure the comprehensibility of each item and appropriate interview duration.

### Data Analysis

The sampling units of the multistage randomized cluster sampling previously mentioned are stratified to reduce study design effects. Municipalities are stratified by size where allocation is proportional to the population of each corresponding municipality, and caregivers are stratified by sex. Data cleaning and aggregation of variables are conducted in accordance with the study objectives.

Every wave undergoes two kinds of analyses: (1) a cross-sectional analysis which includes descriptive and associative analysis between dependent and independent variables (Tables 3 and 4), using bivariate and multivariate logistic regression, and (2) a longitudinal analysis using multilevel analysis. Analysis with a gender perspective is also conducted. All analyses are performed in SPSS (IBM Corp).

Bivariate analysis is used to determine the prevalence of each dependent variable in relation to the independent ones. The respective associations are analyzed by logistic regression analysis with adjustment for age. Multivariate logistic regression analysis with calculation of odds ratios is performed to determine the likelihood of poor health (or another situation of quality of life) according to caregiving characteristics.

A combined model including men and women caregivers with adjustment for all other variables was built to analyze the association between sex and health or quality of life. Forward stepwise selection is used to add variables shown to be significant in the bivariate analysis and other relevant variables from the theoretical model. The same variables are used to build separate models for men and women caregivers to explore factors associated with the studied dependent variable. The magnitude of association in the 3 models is estimated using odds ratios with a CI of 95%. Variance inflation factor analysis rules out multicollinearity between the variables.

**Table 3:** Three categories of dependent variables: (1) health and health-related quality of life, (2) other dimensions of quality of life, and (3) economic value of informal care.

Category and dependent variables		Description
**Variables related to health and health-related quality of life**		
	Health-related quality of life	EuroQol: EQ-5D-DL is a self-assessed questionnaire comprised of a 5-component scale including mobility, self-care, usual activities, pain/discomfort, and anxiety/depression [35]
	Perceived health	Likert: bad, fair, good, very good, and excellent
	Deterioration of health because of care	Yes/no
	Experiences a chronic disease	Yes/no, number and type of chronic diseases experienced
	Mental health	GHQ-12^a^ scale [38]
	Lifestyles	Presenting unhealthy behaviors in at least one of the following factors: alcohol consumption, smoking, hours of sleep, physical activity, and diet
	Burden	Zarit Caregiver Burden Scale validated for use in the Spanish population, consists of 22 questions and 5 possible answers scored from 1 to 5, which establishes 12 different degrees of overload depending on the score obtained: without overload, light overload, and intense overload [37]
	Use of health care services	Yes/no, number and type of health care service used
	Medication use	Yes/no, number and type of medications, prescribed or not
**Variables related to other dimensions of quality of life**		
	Problems derived from care, in work, economic, leisure, and family life	Yes/no
	Time to care for self, derived from care	Yes/no
Variable related to economic value of informal care		Value according to the willingness to pay for care and value according to the willingness to be compensated for care

^a^GHQ-12: 12-item general health questionnaire.

**Table 4:** Three categories of independent variables: (1) caregiver characteristics, (2) caregiving situation, and (3) support received.

Category and independent variables				Description
**Caregiver characteristics**				
	Sex			Male/female
	Age			Continuous
	Province of residence			Granada/Gipuzkoa
	Place of residence			Rural/urban
	Education level			Without studies: lower than primary, primary: EGB^a^/elementary, high school: FP^b^/BIP^c^/higher high school, superior: university or others
	Employment situation			With paid work or without paid work
	Social class			Five categories according to the occupation and according to the National Classification of Occupations of 1994 [40]
	Health status prior to caregiving perceived			Good (excellent, very good, and good) and deficient (regular and bad)
	Relationship between care recipient and the caregiver			Spouse/partner, child, father/mother, other
	Lives with the care recipient			Yes/no
**Caregiving situation**				
	**Characteristics of the care recipient**			
		Sex	Male/female	
		Age	Continuous	
		Degree of dependency	Moderate dependency, severe dependency, and great dependency	
		Cognitive impairment or behavioral problems	Yes/no	
	**Caregiving characteristics**			
		Type of tasks	Personal care, physical mobility, domestic or accompanying tasks within the home and in use of health services, care related to sickness, and tasks outside the home	
		Intensity	Daily hours of care	
		Frequency	Weekly days of care	
		Duration of care	Years of caring	
		Number of dependent people cared for	Number	
**Support received**				
	Informal support			Unpaid support from family or social environment: yes/no
	Formal support			Formal support received in the last 12 months, financial benefit (FB) or home help (HH): without FB or HH, with FB without HH, without FB and with HH, with FB and HH.
	Perceived social support			DUKE-UNC Social Support Index with 11 items validated for use in the Spanish population: low support if score ≤ 32, high social support if score >32 [36].

^a^EGB: Educación General Básica.

^b^FP: Formación Professional.

^c^BIP: ___.

To explore both how individual and group level factors influence trends observed over time, the combination of a longitudinal analysis using multilevel methods is used. The effect of the independent variables on each of the dependent variables is done by means of generalized linear mixed models of a binomial response when the dependent variable is qualitative, or Gaussian when the dependent variable is quantitative. This methodological approach allows for both the analysis of temporal variability in the measurements and heterogeneity among individuals, incorporating random effects into the model coefficients.

A contingent economic evaluation is performed as part of phase II, of which the methodology can be found in other papers from our research group [41].

### Ethical Considerations

The CUIDAR-SE study is conducted according to the guidelines outlined by the Declaration of Helsinki and the law for the protection of patients’ rights (Law 15/2002). Clinical data are not collected as part of this study. Data collection procedures ensure confidentiality, with participant identifiers encrypted using numerical codes managed solely by the research team. These measures are implemented in strict accordance with the data protection laws and regulations in force (Organic Law 3/2018 of December 5, Protection of Personal Data and guarantee of digital rights). Ethical approval was granted by the Research Ethics Committee of Granada (Andalusia [Acta 2/2020]) and the Research Ethics Committee of Euskadi (Basque Country [PI2020068]). Participants provide their written informed consent to participate in the study and are assured anonymity and confidentiality. All data exchanges adhere to the most up-to-date European Union and national data protection regulations.

## Results

The CUIDAR-SE study received funding for each phase of the study starting with phase I in January 2013, phase II in January 2017, and phase III in January 2021 to fund the ongoing 10-year project. Data collection started in 2013 and has continued every year, excluding 2016 due to financial constraints and 2020 due to the COVID-19 pandemic. Total enrollment for the CUIDAR-SE study as of March 2024 stands at 1294 participants. Data collection for the year 2023 is ongoing. There has been ongoing data analysis, initiated after the first wave of data collection in phase I, which investigates gender disparities in caregiver health, quality of life, burden, perceived needs and services, and support received. Results from the first phase have been published [21,41-44]. Currently, work is underway on the data collection of phase III, the data analysis of phases II and III, and on the longitudinal analysis of all phases.

## Discussion

### Contributions of the CUIDAR-SE Study

The CUIDAR-SE study is a pivotal research initiative that will delve into the multifaceted world of caregiving in Spain. With a rigorous longitudinal approach that encompasses 3 waves of data collection at 12-month intervals, for 3 different phases, the CUIDAR-SE study provides the opportunity to capture the evolving experiences of caregivers. Starting in 2013, against the backdrop of the 2008 economic crisis and later with the global COVID-19 pandemic, this temporal perspective enables a comprehensive understanding of caregiving impacts that appear with time within the context in which it occurs. Another distinctive feature of the CUIDAR-SE study is its commitment to incorporating a gender perspective in its design and analysis. This emphasis extends beyond sex-disaggregation of data, to a holistic integration of gender considerations, setting it apart from previous research endeavors in this domain and in Spain. By facilitating gender-based comparisons, the study allows for differential experiences of women and men caregivers to be captured, adding depth to the understanding of caregiving dynamics. Moreover, the CUIDAR-SE study’s multicenter nature with collaboration between research groups with extensive involvement in the field of caregiving, positions it as a pioneering effort. The variation in caregiving contexts between the Andalusia and Basque Country regions allows for a more complex understanding of how different social norms, economic structures, and varying social service support systems influence caregiving.

While other longitudinal studies exist in this field, our study enhances the value of longitudinal design by integrating a gender perspective and fostering multicenter collaboration. This approach provides unique insights into the interplay of major societal events, regional contexts, and gender dynamics, thereby enriching the understanding of caregiving experiences and caregivers’ roles more broadly.

### Limitations

The CUIDAR-SE study, although primarily quantitative in nature, recognizes the value of incorporating qualitative methodologies to delve deeper into the impact of crises and social norms on caregiving. Past endeavors by the research team have involved the use of qualitative methods within this domain [45]. This experience underscores the team’s commitment to continuing this methodological integration in future studies, with the aim of augmenting the findings of the CUIDAR-SE investigation and mitigating this methodological limitation. The prospect of incorporating mixed method approaches in future research endeavors holds substantial potential for enriching discourse within this field.

An important constraint inherent in longitudinal studies is that of lower-than-expected recruitment rates. Such limitations can impede the study’s capacity for conducting comprehensive analyses and may introduce survival bias, particularly prevalent in studies of extended duration. To address this concern, the CUIDAR-SE study has used corrective measures, including a comparative examination of baseline characteristics between participants lost to follow-up to those retained within the study cohort. In addition, recruitment efforts during the inception of the CUIDAR-SE II phase aimed to replenish the participant pool with individuals with comparable characteristics to those recruited at the study’s outset. We emphasize for future longitudinal studies of a long duration to prioritize not only a substantial initial sample size but also to consider budget allocations initially to accommodate potential recruitment challenges.

Participant recruitment, conducted through registration in the Primary Care Health District and Social Services Registry of Granada and Gipuzkoa, respectively may introduce variances in baseline characteristics. Methodological efforts are undertaken during the analysis phase to ensure the comparability of participants from the different regions, thereby homogenizing the study population. This is achieved by considering factors such as the degree of dependency of the person receiving care, the financial support received, and various sociodemographic characteristics of caregivers.

Another limitation of the CUIDAR-SE study is that all participants are solely registered caregivers. However, it is posited that individuals who opt not to register with the corresponding health district or social services registry likely allocate minimal time to caregiving or are of lower caregiving intensity. Thus, the caregiver profile in our study is that of a long-term caregiver providing high-intensity care. Extrapolation of future study results should be to caregivers with a similar profile, who we believe should be the priority target for support interventions.

### Conclusions

The CUIDAR-SE study offers a thorough exploration of caregiving, using a longitudinal design across 3 waves of data collection. Its focus on gender dynamics and collaboration between regions provides valuable insights into caregiving experiences. While recruitment limitations exist, the study’s emphasis on long-term caregivers highlights the need for crucial support interventions. Ultimately, CUIDAR-SE stands as a pioneering effort aimed at addressing the intricate facets of caregiving, not only within Spain, but also in other countries where caregivers’ health and quality of life are similarly impacted.

## Abbreviations

LTC: long-term care
OECD: Organisation for Economic Co-operation and Development

## Data Availability

The data sets generated during and analyzed during this study are available from MMGC main researcher of the CUIDAR-SE Study.
